# Promoting Diabetic Wound Therapy Using Biodegradable rhPDGF-Loaded Nanofibrous Membranes: CONSORT-Compliant Article

**DOI:** 10.1097/MD.0000000000001873

**Published:** 2015-10-30

**Authors:** Cheng-Hung Lee, Kuo-Sheng Liu, Shang-Hung Chang, Wei-Jan Chen, Kuo-Chun Hung, Shih-Jung Liu, Jong-Hwei S. Pang, Jyuhn-Huarng Juang, Chung-Chuan Chou, Po-Cheng Chang, Yi-Ting Chen, Fu-Shing Wang

**Affiliations:** From the Division of Cardiology, Department of Internal Medicine (C-HL, S-HC, W-JC, K-CH, CC-C, P-CC), Department of Thoracic and Cardiovascular Surgery, Chang Gung Memorial Hospital-Linkou, Chang Gung University College of Medicine (K-SL), Department of Mechanical Engineering (S-JL), Graduate Institute of Clinical Medical Sciences, Chang Gung University (J-HSP), Division of Endocrinology and Metabolism, Department of Internal Medicine, Chang Gung University and Chang Gung Memorial Hospital, Tao-Yuan (J-HJ), Department of Biomedical Sciences, College of Medicine, Chang Gung University, Taoyuan (Y-TC), and Shui-Mu Foundation of Chemistry, National Tsing Hua University, Hsinchu, Taiwan (F-SW).

## Abstract

The nanofibrous biodegradable drug-loaded membranes that sustainably released recombinant human platelet-derived growth factor (rhPDGF-BB) to repair diabetic wounds were developed in this work.

rhPDGF-BB and poly(lactic-*co*-glycolic acid) (PLGA) were mixed in hexafluoroisopropyl alcohol, followed by the electrospinning of the solutions into biodegradable membranes to equip the nanofibrous membranes. An elution technique and an enzyme-linked immunosorbent assay kit were used to determine the rhPDGF-BB release rates in vitro and in vivo from this membrane. Eighteen Sprague-Dawley streptozotocin-induced diabetic rats were randomized into 3 groups: rhPDGF-BB-loaded nanofibrous membrane group, PLGA only membrane group, and conventional gauze sponge group for the wound associated with diabetes of rat in each group.

The nanofibrous biodegradable membranes released effective concentrations of rhPDGF-BB for over 21 days. The nanofibrous rhPDGF-BB-loaded PLGA membranes contained more water and were further hydrophilic than PLGA only fibers. The rhPDGF-BB-loaded PLGA membranes considerably helped the diabetic wounds repairing. Furthermore, the proliferative cells and angiogenesis of rats associated with diabetes by rhPDGF-BB-loaded nanofibrous membranes were greater than those of other groups, owing to the increased matrix metalloproteinase 9.

These biodegradable rhPDGF-BB-loaded membranes were effective in treating diabetic wounds as very advanced accelerators during the initial phases of wound-healing process.

## INTRODUCTION

Impaired diabetic wound healing is the most remarkable and severe complication that may happen in the early phase after damage to the skin.^1,2^ The poor healing of such wounds are also the very common result for hospitalization due to wounds associated with diabetes, and may result in amputation as a consequence of preexisting ulceration, regardless of enhanced standards of wound care.^3,4^ Nonhealing ulcers influence 15% of diabetics at some point in their lifetimes and puts extra burdens on healthcare costs.^5^ The pathological changes for poor-healing diabetic wounds include decreased angiogenesis and reduced proliferation and migration of fibroblasts as well as keratinocytes, which result in delayed epithelialization in those wounds.^6,7^ One of the factors to which diabetic complications are attributed is the loss of the expression of growth factors.^8^ The clinical requirement for locally and sustained release of growth factor treatment to promote the diabetic wound healing involved in angiogenesis and cell proliferation is thus significant.

Platelet-derived growth factor (PDGF), a nearly 25,000 Da dimeric protein that comprises 2 polypeptide chains linked together with disulfide bond, performs its functions by binding to the transmembrane domains of receptor tyrosine kinasess to stimulate division and growth of cell, and it has an important role in the formation of blood vessels.^9–11^ On injury, degranulated platelets release PDGF, which is consequently found in the wound drainage, mainly in the initial stage following the injury. Importantly, reduced appearance of PDGF and its receptors is present in healing-impaired hyperglycemic wounds, revealing that expression of these growth factors is crucial for healing.^12,13^ Recombinant human PDGF-BB (rhPDGF-BB) is generated using recombinant DNA technology, with the insertion of gene for the B-chain into the yeast *Saccharomyces cerevisiae*. The pharmacological activity of rhPDGF-BB is comparable with that of naturally occurring PDGF, and involves the promotion of chemotactic recruitment, the formation of granulation tissue, and the proliferation of cells that are involved in wound healing.^14^ The US Food and Drug Administration has approved rhPDGF-BB gel (becaplermin) for the healing of diabetic neuropathic foot ulcers.^15^ However, the gel must be applied once daily, causing discomfort to patients and requiring a considerable nursing effort.

Poly(lactic-*co*-glycolic acid) (PLGA) is the delivery of therapeutic and diagnostic agents that has been widely used due to both biocompatibility and biodegradability. A composite wound covering that is composed of a material coated with antibiotics and a permeable PLGA scaffold is both occlusive and biodegradable.^16,17^ Such a dressing protects the wound when it is no longer necessary to provide needed support, following degradation of hydrolysis to produce nontoxic water and carbon dioxide carried out by dissolving in water. Moreover, the adding of pharmaceutics in PLGA matrices raises the water content of the nanofibers by electrospinning and causes it to act as a great scaffold, promoting the growth and proliferation of cells.^18^ This fact provides a new reason for the application of PLGA with rhPDGF-BB in the management of wounds associated with diabetes.

In our previous work, novel metformin and glucophage/collagen-loaded nanofibrous membranes were developed to present sustainable drugs for repairing wounds associated with diabetes.^19,20^ This work develops nanofibrous biodegradable, rhPDGF-BB-loaded membranes with PLGA using electrospinning to dress wounds associated with diabetes. Topical treatment with a PLGA membrane combined with rhPDGF-BB is hypothesized to promote angiogenesis, epithelialization, and cell proliferation and to support the closure of diabetic wound. Scanning electron microscope (scanning electron microscopy [SEM]) was used to evaluate the electrospun nanofibrous morphology following electrospinning process. The electrospun membranes were examined for the release*s* in vitro and in vivo behaviors of rhPDGF-BB. The effectiveness of the drug-loaded membranes for diabetic wounds healing was also examined.

## MATERIALS AND METHODS

### Materials and Electrospinning

The Resomer RG 503 PLGA 50: 50 polymer with an inherent viscosity midpoint of 0.4 used herein was purchased from Boehringer Ingelheim (Germany). rhPDGF-BB and hexafluoroisopropyl alcohol were obtained from Future Health Biotechnology (Beijing, China) and Sigma–Aldrich (Saint Louis), respectively.

rhPDGF-BB-loaded PLGA and PLGA only membranes were prepared by electrospinning. The laboratory setup for electrospinning involved a hypodermic syringe needle (an inside diameter 0.42 mm) connected to high-voltage (35 kV) direct current (4.16 mA) power supply, and a grounded aluminum collector.^21^ rhPDGF-BB-loaded membrane (PLGA, 0.28 g; rhPDGF-BB, 0.005 g) and PLGA only membrane (PLGA, 0.28 g) as the materials were firstly mixed in 1 mL hexafluoroisopropyl alcohol. The mixtures were then electrospun through the syringe pump (volume velocity, 3.6 mL per hour), to yield thicknesses of about 200 μm. The voltage was applied at 17 kV across the distance of 12 cm between the needle tip and the ground collector. Finally, the nanofibers were removed from the collector and placed in the oven overnight at room temperature (24–26 °C).

### Structure Observation and Analysis – Scanning Electron Microscope (SEM)

Hitachi S-3000N SEM (Tokyo, Japan) was used to obtain the surface structure of nanofibers by electrospinning after the samples had gold coating. The mean fiber diameter of each sample was obtained from the SEM image using Image J software. Measurements of diameter were made at 100 random positions, and the mean of these measurements was taken as the diameter of the nanofibers.

By dividing the mass by its volume, the densities of the electrospun nanofibers were determined. The porosity of electrospinning nanofibrous membrane was analyzed using equation as 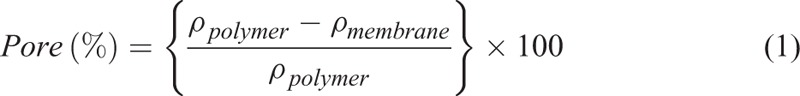


where ρ is the density.

### Mechanical Characteristics of Materials

Under the ASTM D638 standard, a Lloyd tensiometer (AMETEK, PA, USA) with 100 N load cells was prepared to measure the mechanical characteristics of nanofibrous electrospun membranes. The elongation at break and tensile strength were determined as shown in the following equation. 
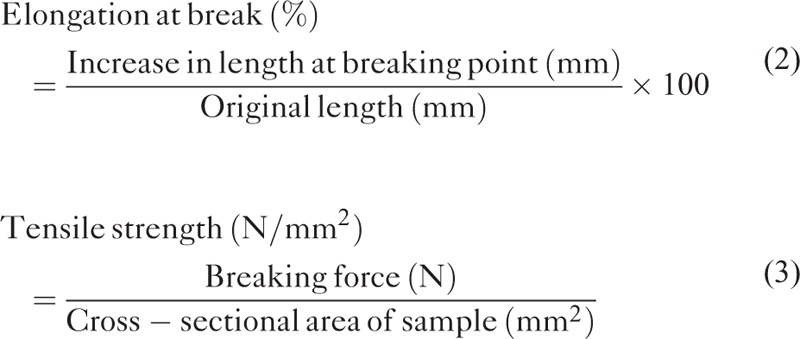


### Contact Angle and Wetting Properties

The water contact angle measurement uses an analyzer (First Ten Angstroms, USA) to determine the contact angles of water on the nanofibrous membranes surface. Membranes with an area of 10 mm × 10 mm were prepared and fixed on the test plate after dropping distilled water onto the surfaces. Half automatic video-based measurements of contact angle of the rhPDGF-BB-loaded and the PLGA only membranes and averaged for each liquid were performed at 25 °C.

### Water Absorption and Retention Capacity

The water absorption and retention capacities of membranes were evaluated at regular intervals of 0.5, 1, 2, 3, 8, and 24 hours. The accurate content of water in each sample was defined as percentage of water content. 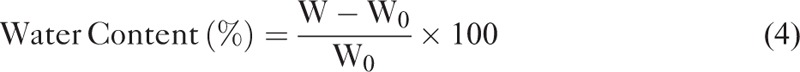


where W_0_ is the weight before submersion in water and W is the after submersion in water.

### Wound Repairing in Rats Associated with Diabetes

The preliminary experiments showed mean values of percentage wound closure on day 14 were about 11% and 15% in treatment and control groups, respectively. The observed standard deviation was about 2%. Given the α of 0.05 and desired power of 0.9, the calculated minimal sample size was 6 in each group. Eighteen Sprague-Dawley rats, weighing 368 ± 42 g, were prepared at the beginning of experiments. All procedures related to animal care were institutionally approved (Chang Gung University Animal Research Committee), and all of the rats were prepared in accordance with applicable regulations of the Ministry of Health and Welfare of Taiwan. All the study animals were separately kept in cages and maintained in a room at controlled temperature and humidity with a fixed 12 h artificial light period; where they were allowed free access to water and food (standard rodent chow) – both before starting and during the period of the experiment. Diabetes was made in rats using a single intraperitoneal injection of sterile streptozotocin (Sigma) (70 mg/kg of body weight) with 100 mM sodium citrate buffer at pH 4.5 in 0.1 M NaCl. Seventy-two hours of development of diabetes, the rats with plasma glucose more than 300 mg/dL were considered as diabetic rates and used for experiments. After 1 week of streptozotocin injection, the subsequent studies were carried out.

For each rat, a sterile disposable template (diameter, 8.0 mm) was placed on middle back of both sides, and full-thickness excision wounds were created following anesthetization. Eighteen rats were randomly separated into 3 groups: group A, the rhPDGF-BB-loaded nanofibrous membrane; group B, PLGA only membrane; and group C, a conventional gauze sponge as controls was treated to the wound associated with diabetes of rat in each group. No extra topical pharmaceutics were treated and no dressings were changed during the period of healing.

In this study, a glass side was traced onto the wound edges to calculate wound area and was utilized to determine wound area. After wounding, the area defined using the trace was acquired on days 0, 3, 7, and 14 and were expressed as square mm.

In rats of 3 groups, whole blood glucose concentration was measured daily in blood obtained from the tail vein by OneTouch blood glucose strips (LifeScan, Milpitas). Insulin treatment was injected by Sanofi-Aventis glargine (Frankfurt, Germany) if the rat had “high” glucose readings and weight loss.

After 3, 7, and 14 days of healing, an excising wound down to the fascia along with 0.5 cm edge of unwounded dorsal skin was prepared on each entire wound. Optimal cutting temperature compound is used to embed tissue samples prior to frozen sectioning with a cryostat microtome.

Intradermal tapping below the dressings was aspirated with 19-gauge needle for in vivo release analysis, on the rats to whose wounds were treated rhPDGF-BB-loaded membranes on days 7 and 14.

### The Release of In-Vitro and In-Vivo RhPDGF-BB

Active rhPDGF-BB concentrations were obtained using Quantikine PDGF-BB Enzyme-linked immunosorbent assay kits (R&D Systems, Minneapolis). Samples were analyzed on a multiple detection plate reader (Tecan SAFIRE, Durham) at a wavelength of absorption of 450 nm using a reference wavelength of 570 nm. The standards in each kit were used to plot standard curves.

All samples were carried out at least 3 times, and the sample dilutions were preformed to get the unidentified concentrations from the range of the standard assay curve. All measures (correlation coefficient > 0.99) were plotted as a calibration curve.

The in vitro release properties of the rhPDGF-BB-loaded nanofibrous membranes were evaluated by an elution method. The electrospun membranes (diameter, 8 mm) were put in a test tube (glass) (n = 3) with 0.15 mol/L at pH 7.4 phosphate buffer solution (PBS) (1 mL). The test tubes were incubated at 37 °C for 1 day prior to collect and analyze the eluent. Fresh PBS (1 mL) was then renewed every day for 3 weeks. The in-vivo rhPDGF-BB concentrations of the excised samples were as well obtained using the enzyme-linked immunosorbent assay. All in-vivo experiments (n = 5) were determined following dilution with PBS and were calculated by the standard curve.

### Immunofluorescence

The chemical reagents were acquired from Sigma, Molecular Probes Inc. (Eugene). Prior to being frozen sectioned by a microtome-cryostat, the tissue samples were fixed in optimal cutting temperature. The tissue sections were visualized by primary antibodies against matrix metalloproteinase 9 (MMP 9) (Abcam, Cambridge), secondary Cy3-conjugated antibody (Chemicon, Temecula), and DAPI-staining (blue) (n = 3).

### Statistics and Data Analysis

Data are shown as mean and standard deviation when normal distribution is found using a Kolmogorov–Smirnov test. One-way ANOVA was performed to identify statistically significant variations among data. To make multiple comparisons, the post-hoc Bonferroni method was used to find substantial differences between pairs of groups. If the *P* value was less than 0.05, differences were considered statistically significant. The collected data were performed and analyzed using SPSS version 17.0 (SPSS Inc., Chicago, IL).

## RESULTS

### Fabrication and Characterization

**FIGURE 1:**
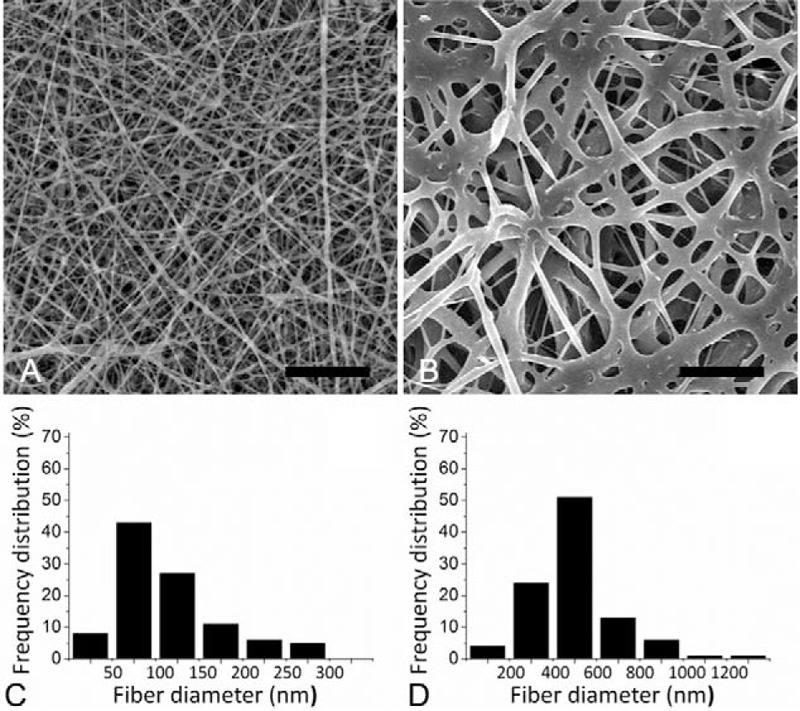
SEM pictures for electrospun nanofibers; (A) rhPDGF-BB-loaded PLGA, and (B) PLGA only. (Scale bar: 5 μm). °). PLGA = poly(lactic-*co*-glycolic acid, rhPDGF-BB = recombinant human PDGF-BB.

Figure 1 shows the SEM images of the electrospun nanofibrous mesh (3000×). rhPDGF-BB-loaded PLGA nanofibers (Fig. 1A) exhibited a narrower distribution of diameters than did PLGA only fibers (Fig. 1B). The diameters of the nanofibers with rhPDGF-loaded PLGA and PLGA only were 116 ± 60 nm and 494 ± 190 nm, respectively. The porosities of the nanofibrous membranes were similar (76.1 ± 1.2% for the rhPDGF-BB-loaded PLGA and 77.0 ± 1.7% for the PLGA only nanofibers).

**FIGURE 2:**
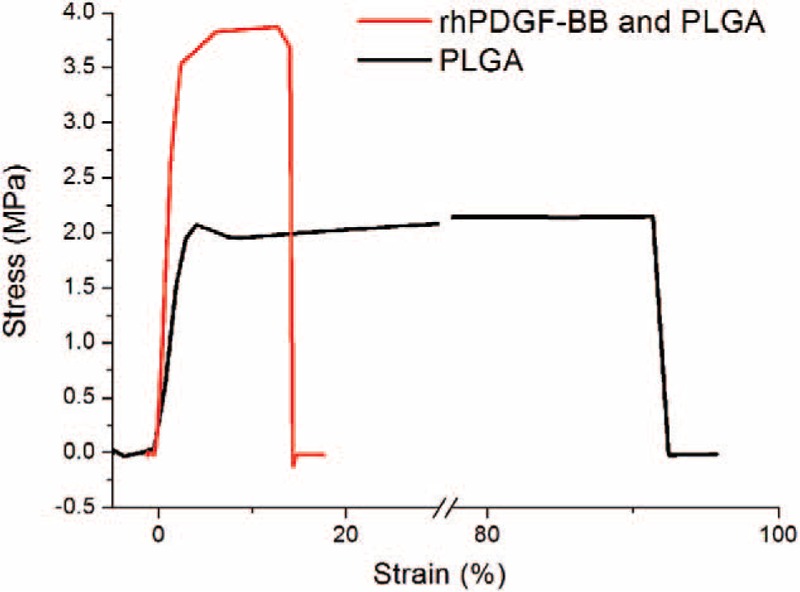
Stress–strain curves of nanofibers. Top trace is for group A, showing a elongation at breakage of 13.8% and tensile strength of 3.87 N/mm^2^. Bottom trace is for group B with elongation at breakage of 89.0% and tensile strength of 2.14 N/mm^2^.

The results of mechanical characteristics of the nanofibrous electrospun membranes in Figure 2 indicate that the rhPDGF-BB-loaded nanofiber hold a larger tensile strength than the PLGA only (4.27 ± 0.59 vs 1.94 ± 0.35 N/mm^2^, respectively) (*P* < 0.05). However, the PLGA only nanofibers displayed greater elongation at break (89.2 ± 7.3%) than did the rhPDGF-BB-loaded nanofibers (10.7 ± 3.0%) (*P* < 0.05).

**FIGURE 3:**
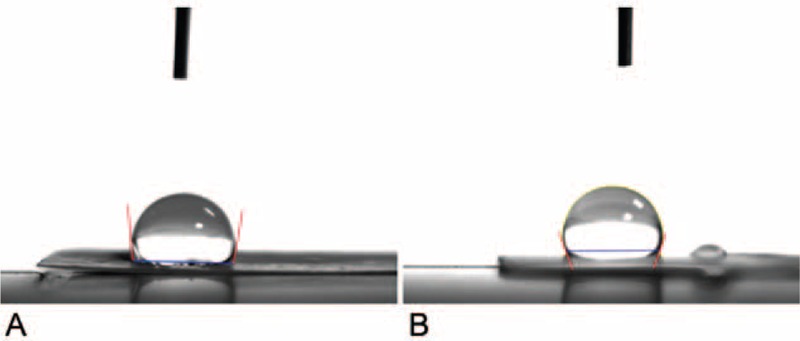
Contact angles of water. (A) rhPDGF-BB-loaded PLGA (94.5°), and (B) PLGA only nanofibers (110.3°). PLGA = poly(lactic-*co*-glycolic acid, rhPDGF-BB = recombinant human PDGF-BB.

Figure 3 presents contact angles of water for the rhPDGF-BB-loaded and the PLGA only nanofibrous membranes were 92.5° ± 2.6° and 107.3° ± 3.1°, respectively. Obviously, addition of rhPDGF-BB to PLGA greatly decreased the hydrophobicity of the nanofibrous electrospun membranes (*P* < 0.05).

**FIGURE 4:**
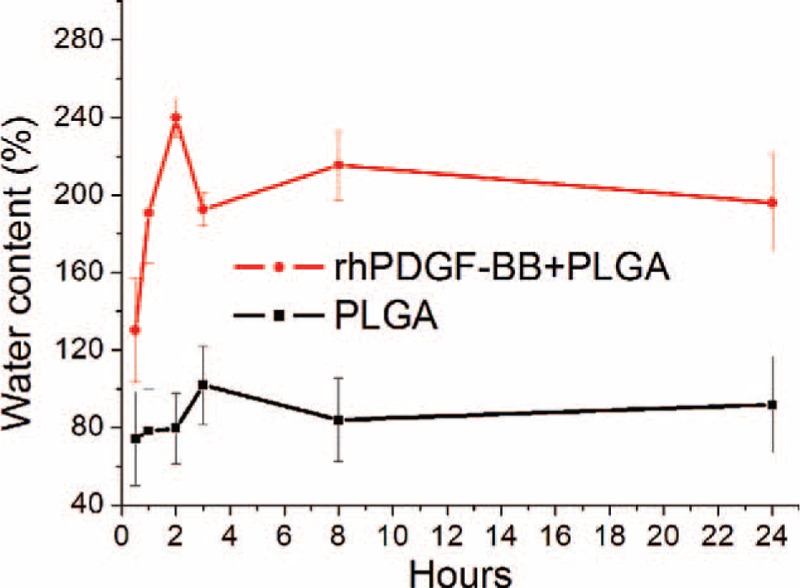
Various water content in nanofibrous membranes at the different time.

The consequences of changes of the water content in Figure 4 indicate that the content capacity of water in the electrospun nanofibrous membranes had raise overtime. The nondrug-loaded membranes got the maximum water content level (102 ± 20%) at 180 minutes, whereas the rhPDGF-BB-loaded nanofibers got their maximum level of water content of 240 ± 10% at 120 minutes. The ability of the PLGA only nanofibers to embrace water was less than that of rhPDGF-BB-loaded (ANOVA *P* < 0.05).

### The Release of rhPDGF-BB

**FIGURE 5:**
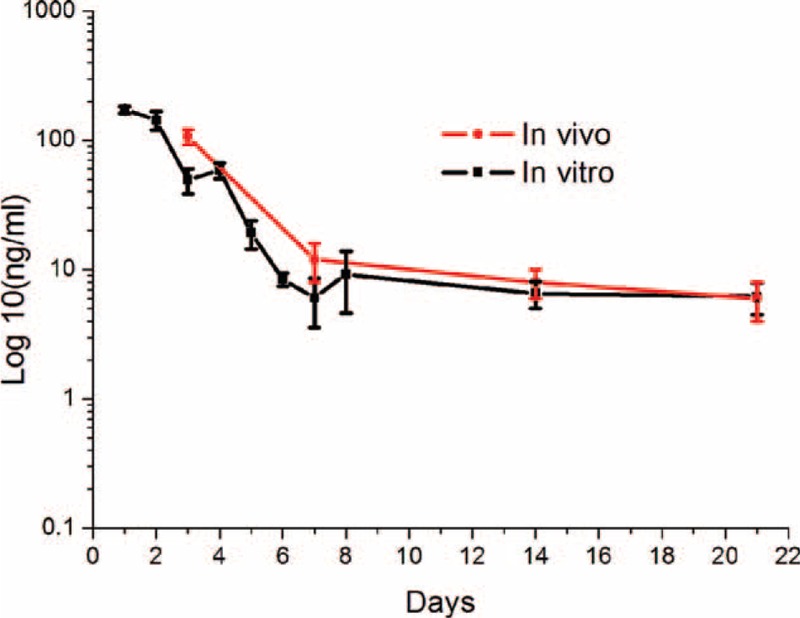
In vitro and in vivo release of recombinant human PDGF-BB (rhPDGF-BB).

Figure 5 shows the daily characteristics of released rhPDGF-BB in vitro. The rhPDGF-BB-loaded nanofibers constantly delivered rhPDGF-BB for 21 days, with a high initial surge period of 48 hours (>144 ng/mL). A next highest rate of release was noted on 96 hours (>59 ng/mL), following which the concentration gradually decreased, and a constant rate of release was detected on days 14 to 21 (around 6 ng/mL).

In-vivo rhPDGF-BB pharmaceutical levels were evaluated on days 3, 7, 14, and 21 shown that the concentration had highest level on 72 hours (107 ± 14 ng/mL), subsequently that the accumulative concentration decreased.

### Wound Repairing, Histopathological Findings, and Immunofluorescence of MMP-9

**FIGURE 6:**
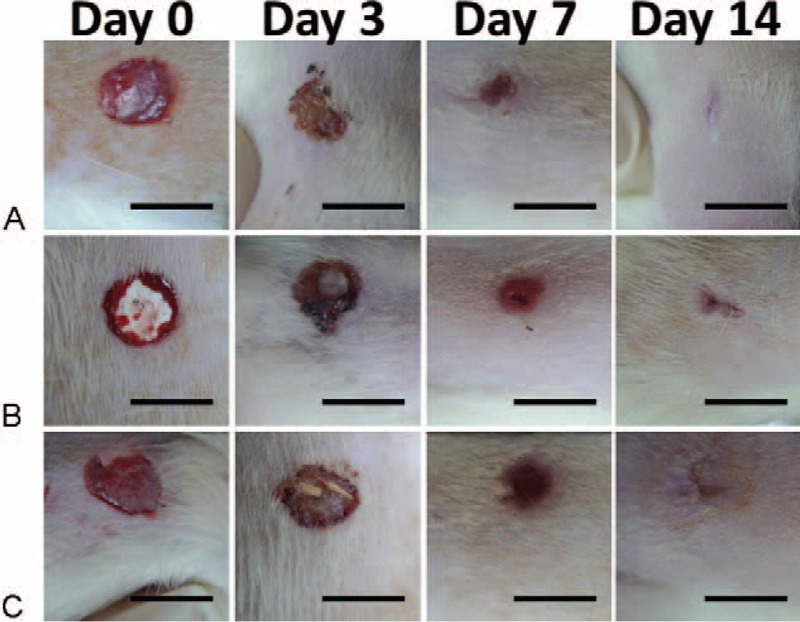
Rounded diabetic wounds of 3 groups on various days after treatment. (Scale bar = 10 mm).

**FIGURE 7:**
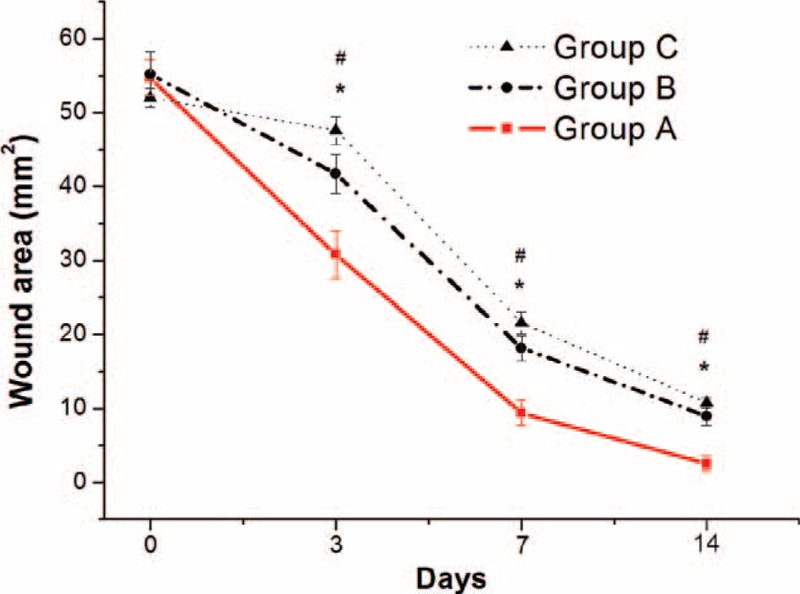
The repair of wound of 3 groups. (^∗^Group A vs group B, *P* < 0.05; ^#^group A vs group C, *P* < 0.05, post-hoc analysis).

Figure 6 presents the gross appearance of the wounds in the animals from each group on various days (0, 3, 7, and 14) following healing. The wounds in rhPDGF-BB-loaded group closed obviously quicker than other 2 groups on each of those days. On these days, the zones of the wounds in control groups were comparable. Figure 7 shows the areas of the wounded skin following different days of repairing. Whereas the regions of the wounds that had been sheltered by PLGA only membrane and gauze declined slowly to 9.0 ± 1.3 mm^2^ and 10.8 ± 0.7 mm^2^, respectively, following 2 weeks, the regions of the wounds that were sheltered by the rhPDGF-BB-loaded membranous were only 2.5 ± 1.0 mm^2^ on day 14. The use of rhPDGF-BB-loaded electrospun nanofibers (group A) upgraded wound repairing above that treated in both control groups. (post-hoc *P* all < 0.05).

**FIGURE 8:**
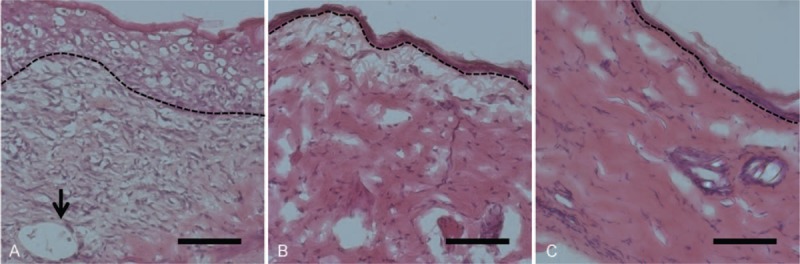
The images of histological section on day 14 after treatment. (Scale bar = 100 μm). Greater infiltration of proliferative cells and vascularization with lining endothelium (arrow) were observed in group A than in other groups.

The histological pictures are displayed in Figure 8. The nanofibrous scaffoldings were thoroughly incorporated into the nearby skin in both membrane groups without a significant inflammatory response. The pictures reveal that PLGA with rhPDGF-BB-loaded nanofibers enhanced skin recovery better than that did using other 2 treatments. On day 14, the amount of proliferative cells was higher in group A than in other groups. Additionally, the rhPDGF group demonstrated significant neovascularization and the lining of endothelial cells. Fourteen days following operation, the wounds in all 3 groups had nearly repaired, as covering scattered inflammatory cells and newly integrated fibrous tissue in the subcutis and dermis by an entirely re-epithelialized epidermis. Nevertheless, the wounds that were preserved with rhPDGF-BB-loaded nanofibers (group A) presented a denser stratum corneum than those in other groups.

**FIGURE 9:**
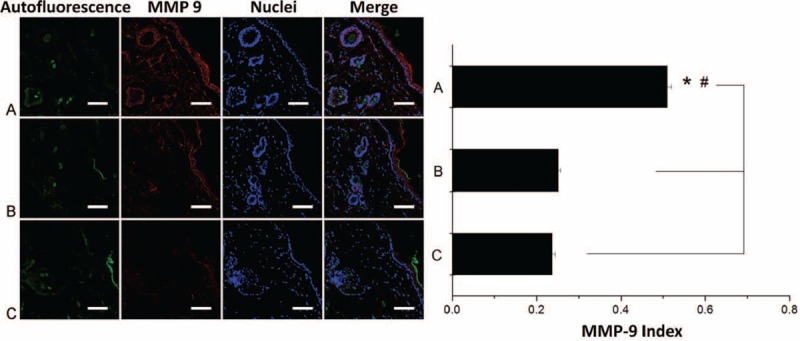
Effects of matrix metalloproteinase 9 (MMP-9) content on day 14. (Scale bar represents 75 μm). (^∗^Group A vs group B, *P* < 0.05; ^#^group A vs group C, *P* < 0.05, post-hoc analysis).

The ratio of the staining density of MMP-9 and DAPI-labeled nuclei in the dermis was analyzed as the labeling index of interstitial MMP-9 (Fig. 9). On day 14, both control groups had significantly less amount of MMP-9 than did those in the rhPDGF-BB-loaded membranes group (group A 0.51 ± 0.01; groups B 0.25 ± 0.01; and group C 0.24 ± 0.01) (post-hoc *P* < 0.001).

## DISCUSSION

Angiogenesis is disturbed in diabetes, decelerate wound repairing and weaken immune activity, together of which are crucial to endurance and salvage from major skin injury.^22–24^ Hyperglycemia is also a threat reason for epithelial downgrowth resulting skin damage.^25^ Cheng et al^26^ and Kobayashi et al^27^ demonstrated molecular mechanisms of PDGF-BB that promote cell proliferation and revascularization and improve dermal wound healing in diabetic models.

The biodegradable nanofibrous rhPDGF-BB-loaded membranes released high and effective concentrations for over 21 days in vitro and in vivo to help diabetic wounds heal herein. The histopathological pictures of cell proliferation and angiogenesis in the wound regions revealed that rhPDGF-BB-loaded membranes provided more active diabetic wounds repair than did nanofibers only or gauze dressings. rhPDGF-BB-loaded biodegradable scaffold was functionally effective in treating wounds associated with diabetes and was applicable as accelerators during the wound-healing process.

Scaffoldings that are used for tissue regeneration purposes should have mechanically properties for supporting cellular morphogenesis. The tensile strength of electrospun nanofibers can be improved with the addition of rhPDGF. Despite the elongation at break is reduced, the rhPDGF-BB-loaded nanofibrous membranes still exhibit an extensibility of 15%, which provides advantages in terms of comply with the tissue shrinkage during wound healing.

Electrospun drug-loaded nanofibers had a narrower distribution of diameters than did the PLGA only nanofibers, mainly owing to the presence of rhPDGF-BB. With the addition of rhPDGF-BB, the polymer content in the solution was reduced. It is thus easier for the electrostatic repulsion force to stretch the fibers during electrospinning. The diameters of electrospun drug-loaded nanofibers decreased accordingly. Despite the fact that the width of the rhPDGF-BB-loaded nanofibers that were developed herein was smaller than the reported optimal diameter,^28^ the developed nanofibrous carriers supported accelerated wound repair and promising cell proliferation, perhaps because rhPDGF-BB in the electrospun textiles supported the formation of blood vessels.

Electrospinning is regarded as an efficient method for fabricating nanofibrous polymers, leading to widely utilized PLGA scaffolding systems for tissue engineering as a fundamental material. Numerous polymers, including PLGA ultrafine fibers, have been effectively developed by electrospinning in current years, generally in solution and sometimes in melt method. The novel extracellular matrix that mimics a nonwoven microfibrous arrangement in electrospun PLGA structures for use as tissue engineering have been fully investigated.^29^ In this work, the rhPDGF-BB-loaded nanofibrous PLGA membranes are highly porous with favorable mechanical strength, flexibility, and extensibility, making feasible their practice in coverings allowing skin contraction during the healing course.

Wound repair usually progresses more slowly under dry surroundings than under moist settings.^30^ Conventional wound healing dressing fabrics sometimes absence any water-holding characteristics to reduce dehydration. A perfect wound dressing fabrics should be able to confine the wound humidity and wetness; it should be penetrable to CO_2_ and oxygen, and so enhance repair of wound. Increasing the outward hydrophilicity of hydrophobic fabrics supposedly increases cell migration and adherence, and cell proliferation in particular. Cell-holding biomolecules can be adsorbed competitively by outsides with adequate humidity, causing favoring cell contact and attachment.^31^ The PLGA only nanofibers herein had relative hydrophobicity, the rhPDGF-BB-loaded nanofibers had greater hydrophilicity. Hence, controlling superficial hydrophilic property of PLGA scaffoldings will be favorable for cell proliferation. Furthermore, rhPDGF-BB-loaded nanofibers also show the peak level of water content (at day 2) earlier than PLGA only nanofibers (at day 3), mostly because of the manifestation of hydrophilic rhPDGF-BB. Scaffoldings with high levels of water contents at earlier days should be able to achieve improved wound healing.

Decreasing porosity and pore size of the membrane with smaller fiber diameter is thought to have less amount of air pocket phase at the membrane surface, augmenting the hydrophilic environment.^32^ The surface roughness or chemistry of the membrane, including diameters, pores content, thickness of the nanofibrous membrane, determines the measurement of static contact angle.^33^ Clearly the water contact angle was reduced substantially with decreasing fiber diameter. Since the PLGA only nanofibers herein had higher water contact angle than that of rhPDGF-BB-loaded nanofibers, mostly owing to the existence of hydrophilic rhPDGF-BB and decreasing fiber diameter. Moreover, the permeable mandatory background offers abundant humidity and supports the sustained release of the rhPDGF-BB, precluding becoming dry of the wound area, and thus supporting wound repair. The carrier also eliminates the requirement for continual wound redressing and cleaning, aiding the body to heal more effectively and diminishing the upset and distress of patients with diabetic wound.^34^

As determined from the characteristics of rhPDGF-BB release in vivo and in vitro, the drugs herein were almost distributed in the mass of the PLGA carrier by electrospinning. However, some of the drugs may have been present on the nanofibrous surfaces, causing the first surge of pharmaceutical release. Thereafter, polymer degradation and diffusion alone controlled the drug release. A fairly continual drop in the release rate of effective dose of rhPDGF-BB was thus observed.^35^ Generally, the biocompatible and biodegradable matrixes with drug loading delivered great amount of rhPDGF-BB for over 21 days in effectively treating wounds associated with diabetes.

Regulation of immune response and repair during wound healing after ischemic injury of tissue depends on angiogenesis, which is the growth of new capillaries from existing ones. PDGF-BB is a well-characterized biologically potent growth factor, which is required for the development of high primitive vascular plexuses density.^36^ PDGF is also required cellular division of fibroblasts, which are connective tissue cells that are especially abundant in healing wounds.^37^ Exogenously administered PDGF has been established to stimulate proliferation, gene expression, and chemotaxis in fibroblasts and monocytes-macrophages, and to increase significantly the migration of fibroblasts and inflammatory cells, enhancing the formation of the collagen and extracellular matrix and thereby diminishing the duration of the repair course.^38^ The discovery that MMPs can reveal cryptic signaling locations on extracellular matrix assembly and stimulate and release matrix containing bound growth factors suggests the involvement of MMPs in angiogenesis.^39^ Genetic MMP-9 deficiency has been found to retard bone growth by inhibiting of angiogenesis.^40^ This work showed that diabetic wound healing is enhanced by the overexpression of MMP-9, which results in angiogenesis with rhPDGF-BB-loaded nanofibrous membranes.

Finally, an enhanced dressing can afford faster wound repair and an improved result following wound-forming injuries, with decreased discomfort, and infection; it also lessens budgets by decreasing the healing interval, and permitting for fewer repeated attention and easier performance by medical specialists. The investigational results in this work indicate that the PLGA nanofibers for the release of rhPDGF-BB had favorable biocompatibility and remarkably augmented wound recovery. This result additionally indicates that the developed rhPDGF-BB-loaded PLGA nanofibrous dressings can serve as an applicable tissue-engineering platform for poor-healing wound by diabetes.

## CONCLUSIONS

The nanofibrous biodegradable drug-loaded membranes that sustainably released rhPDGF-BB to heal diabetes-related wounds were developed herein. This nanofibers delivered effective levels of rhPDGF-BB for over 21 days. The nanofibrous rhPDGF-BB-loaded PLGA membranes contained more water and were greater hydrophilic than PLGA only fibers. The rhPDGF-BB-loaded PLGA membranes considerably supported the repair of diabetic wounds. Additionally, the proliferative cells and angiogenesis of wounds associated with diabetes using rhPDGF-BB-loaded nanofibers were greater than those of control groups, as a result of the increased MMP 9. These biodegradable rhPDGF-BB-loaded membranes were functional and effective in treating diabetic wounds as an advanced accelerator throughout the early phases of wound-healing course.
